# Caffeic Acid Phenethyl Ester Suppresses Adhesion to Mediate Its Antibiofilm Activity Against Methicillin-Resistant *Staphylococcus aureus*

**DOI:** 10.3390/microorganisms14071601

**Published:** 2026-07-22

**Authors:** Kaiyue Feng, Haoni Luan, He Sang, Wenhan Qiu, Rui Yang, Jie Cheng, Wei Feng, Wei Xu, Peng Song, Fei Wang

**Affiliations:** 1 School of Pharmaceutical Sciences and Food Engineering, Liaocheng University, Liaocheng 252059, China; 2 College of Agriculture and Biology, Liaocheng University, Liaocheng 252059, China; 3 Shandong Key Laboratory of Applied Technology for Protein and Peptide Drugs, Liaocheng University, Liaocheng 252059, China; 4 State Key Laboratory of Macromolecular Drugs and Large-Scale Preparation, Liaocheng University, Liaocheng 252059, China

**Keywords:** methicillin-resistant *Staphylococcus aureus*, caffeic acid phenethyl ester, biofilms, adhesion

## Abstract

Methicillin-resistant *Staphylococcus aureus* (MRSA) poses a serious threat to public health and can form biofilms to enhance its drug resistance. Caffeic acid phenethyl ester (CAPE), which is primarily extracted from propolis, possesses diverse biological activities. However, its effect on anti-MRSA biofilms and the relevant mechanisms have not been fully clarified. Therefore, this study explored the ability of CAPE to combat MRSA biofilms. The results showed that CAPE has significant antibiofilm activities against MRSA. The minimum inhibitory concentration (MIC) values of CAPE were 256 µg/mL for the MRSA strains ATCC 33591, CI2, and CI3. Crystal violet (CV) assay and XTT assays demonstrated that CAPE could inhibit the formation and consolidation of MRSA CI2 biofilms. Experiments on scanning electron microscopy (SEM), bacterial adhesion assays, and the levels of extracellular polysaccharides confirmed that CAPE can inhibit bacterial adhesion, as well as the synthesis of extracellular polysaccharides in MRSA CI2. Real-time quantitative PCR (RT-qPCR) experiments confirmed that CAPE can affect the expression of MRSA *icaADBC*, *sarA*, *fnbAB*, and *clfAB* genes. Therefore, the proposed antibiofilm mechanism of CAPE involves the downregulation of aforementioned genes, leading to reduced production of extracellular polysaccharides and adhesion-related proteins, thereby weakening MRSA adhesion and ultimately exerting an antibiofilm effect. In conclusion, these findings suggest CAPE is a promising candidate drug as an antimicrobial agent for managing and preventing biofilm-associated infections caused by MRSA.

## 1. Introduction

Methicillin-resistant *Staphylococcus aureus* (MRSA) is a highly dangerous bacterial species that ranks among the prevalent causative agents responsible for numerous clinical illnesses, including pneumonia, wound infections following surgery, bloodstream infections, and infections of the urinary system [1]. Since its initial identification in the 1960s, MRSA infections have become increasingly prevalent, posing a substantial challenge to public health systems worldwide [2]. The *mecA* gene carried by MRSA encodes penicillin-binding protein 2a (PBP2a), which exhibits markedly reduced affinity for β-lactam antibiotics and mediates intrinsic resistance to β-lactam agents, including penicillins, cephalosporins and carbapenems [3]. Some MRSA strains have even developed resistance to vancomycin, which is regarded as the “last line of defense”, thereby severely complicating the clinical management of MRSA infections [1]. The 2024 surveillance data from the China Antimicrobial Surveillance Network (CHINET) showed that the detection rate of MRSA was as high as 28.4% [4]. All these phenomena indicate that MRSA is becoming an increasingly serious public health threat.

Biofilm is a complex, three-dimensional microbial aggregate formed by microorganisms during their growth, enclosed by extracellular polymeric substances (EPS) secreted by themselves. Its main components include extracellular polysaccharides, proteins, and extracellular DNA (eDNA) [5]. The barrier structure formed by EPS can significantly hinder the penetration of antibiotics. Therefore, compared with planktonic bacteria, bacteria residing within biofilms display much greater tolerance toward antimicrobial drugs and pose greater challenges for eradication [6]. Research data indicate that bacteria within biofilms exhibit 10 to 1000 times higher tolerance to antimicrobial agents compared with their planktonic counterparts [7]. By enhancing bacterial resistance and evading host immunity, biofilms cause various refractory diseases such as medical device-related infections, chronic wound infections, and multi-organ infections. This not only leads to recurrent infections, prolonged treatment cycles, and a sharp increase in medical costs but may also trigger severe complications such as sepsis and infectious shock, significantly increasing patient mortality and posing a major threat to human health [8,9]. In various clinical infections, MRSA biofilm is an important pathogenic factor that mediates bacterial resistance and contributes to infections [10]. Consequently, since MRSA ranks among the primary bacterial pathogens implicated in biofilm-associated infections, there is a pressing need to develop novel therapeutic agents that specifically target its biofilms.

Caffeic acid phenethyl ester (CAPE, Phenethyl 3,4-dihydroxycinnamate, C_17_H_16_O_4_) is a natural phenolic compound widely present in propolis and one of the main components through which propolis exerts its pharmacological activity [11,12]. According to existing research, CAPE exhibits multiple bioactive properties, including antimicrobial, antioxidative, anti-inflammatory, immunoregulatory, and neuroprotective functions, which indicates that it holds considerable promise for diverse medical applications [13]. Recent research has mainly focused on the application of CAPE in the field of anticancer [14,15,16], as well as its application in cell protection and tissue damage prevention [17,18,19]. Additionally, research has also focused on the application of CAPE in nanoscience, such as CAPE-PLGA-NPs and Bet–CAPE, and these studies primarily concentrate on the improvement of preparation processes [20,21]. Nevertheless, CAPE has attracted increasing attention in the field of antibacterial research. Previous research results indicate that CAPE can significantly inhibit the adhesion process of multi-species biofilms associated with dental caries [22]. However, research on the antibiofilm efficacy and underlying molecular mechanisms of MRSA biofilm remains relatively scarce. Therefore, this study aims to conduct an in-depth investigation into the antibiofilm activity of CAPE against MRSA, elucidate its effects and mechanism of action, and provide both a theoretical foundation and empirical evidence to support the creation of novel therapeutic agents targeting MRSA.

## 2. Materials and Methods

### 2.1. Materials and Bacterial Strain

Caffeic acid phenethyl ester (CAPE, purity≥98% by HPLC) was obtained from Shanghai Yuanye Biotechnology Co., Ltd. (Shanghai, China). The tested MRSA strains ATCC 33591 and clinical isolates CI2 and CI3 were maintained in the Natural Product Bioactivity Laboratory at the School of Pharmaceutical Sciences and Food Engineering of Liaocheng University. All bacterial isolates were stored at −80 °C within tryptic soy broth (TSB) supplemented with 20% glycerol.

### 2.2. Determination of Minimal Inhibitory Concentration (MIC)

To determine the lowest concentration of CAPE capable of inhibiting MRSA growth, this study employed the broth microdilution technique for MIC assessment [23]. Briefly, 100 μL of different concentrations of CAPE and 100 μL of MRSA strain (ATCC 33591, CI2, and CI3) suspension were added to a 96-well plate. CAPE was tested within a final concentration range of 16 to 512 µg/mL, and the MRSA bacterial suspension was adjusted to a final concentration of 1.5 × 10^7^ CFU/mL. The negative control contained no CAPE but was otherwise identical to the treatment group. A 10 µg/mL vancomycin hydrochloride solution was designated as the positive control. The plates were incubated at 37 °C for 18 h. Next, each well received 20 µL of resazurin sodium (1 mg/mL), followed by a 3-h incubation period at 37 °C under light-free conditions. The MIC value was defined as the lowest concentration of CAPE at which the color of the solution did not change from purple to orange.

### 2.3. Inhibitory Effect of CAPE on Biofilm Formation in MRSA

The antibiofilm formation experiment was conducted based on established methods [24,25]. Add 100 µL of CAPE and 100 µL of MRSA CI2 suspension to a 96-well plate. CAPE was tested within a final concentration range of 8 to 256 µg/mL, and the MRSA suspension was adjusted to a final concentration of 1.5 × 10^7^ CFU/mL. Tryptic Soy Broth (TSB) supplemented with 1% sucrose was used as the culture medium. Incubation was carried out at 37 °C for 24 h. Once the incubation period ended, all planktonic bacterial cells were removed by suction, followed by three rounds of washing for every well using phosphate-buffered saline (PBS, pH 7.4). The biomass and metabolic activity of MRSA biofilms were assessed employing both the crystal violet (CV) staining method and the XTT reduction assay [26].

### 2.4. Inhibitory Effect of CAPE on Mature Biofilm in MRSA

Conduct anti-mature biofilm experiments on CAPE regarding MRSA [24,25]. First, MRSA CI2 suspension was adjusted to 1.5 × 10^7^ CFU/mL and aliquoted into 96-well plates. TSB supplemented with 1% sucrose was used as the culture medium, and incubation was carried out at 37 °C for 24 h. Subsequently, all non-adherent bacteria were removed by suction, followed by three PBS washes for every individual well. After that, 200 µL of fresh TSB medium containing CAPE at final concentrations ranging from 8 to 256 µg/mL was dispensed into each well. Incubation was carried out at 37 °C for 24 h. Once the incubation was complete, all planktonic bacteria were removed by suction and then washed using PBS. The biomass and metabolic activity of MRSA biofilms were assessed employing both the CV staining method and the XTT reduction assay [26].

### 2.5. Scanning Electron Microscopy Observation of MRSA Biofilm

The method previously reported was adopted with slight modifications to evaluate the effect of CAPE of MRSA biofilms using a scanning electron microscope (SEM) [27]. A sterile coverslip was placed in each well of the six-well plate. Subsequently, the MRSA CI2 suspension (1.5 × 10^7^ CFU/mL) supplemented with 1% sucrose was added. Incubation was at 37 °C for 24 h to establish biofilms. After discarding the supernatant from each well, fresh CAPE solution was added to replace it in the wells, and thereafter, the plates were kept at 37 °C for another 12 h of incubation. The coverslips were washed and then fixed by immersion in 2.5% glutaraldehyde solution overnight at 4 °C. After that, the MRSA biofilms on the coverslips were dehydrated using graded concentrations of ethanol and tert-butanol. Freeze-dry the samples using a freeze dryer (SCIENTZ-10 N/C, Ningbo, China). The samples were coated with gold using sputtering technology and observed under a scanning electron microscope (S-4800, Hitachi, Tokyo, Japan).

### 2.6. Bacterial Adhesion Assay

According to an established method, CAPE’s impact on the adhesion of MRSA was evaluated [28,29]. The procedure was as follows: 100 μL of TSB solution containing CAPE and sucrose was added to each well of a 96-well plate. Subsequently, 100 μL of MRSA CI2 suspension at 3.0 × 10^8^ CFU/mL was added. The final concentration of CAPE in each well ranged from 8 to 128 μg/mL. The sucrose concentration was 1%. Incubation was carried out at 37 °C for different durations. Planktonic cells were removed by aspiration, and each well was washed three times with PBS. Then, each well received 200 μL of TSB medium, after which the plate underwent a 5-min sonication treatment. The OD value at 600 nm was measured using a microplate reader (FlexA-200, Aosheng, Hangzhou, China).

### 2.7. Analysis of Extracellular Polysaccharide Production

The established method was used to investigate the effect of CAPE on the synthesis of extracellular polysaccharides in MRSA CI2 [24]. The specific procedures are as follows: an MRSA suspension was prepared at a final concentration of 1.5 × 10^8^ CFU/mL. This suspension was then mixed with CAPE at final concentrations of 8–128 µg/mL. After that, the sucrose concentration of the system was adjusted to 1%, and the mixture was incubated at 37 °C for 24 h. After incubation, the culture was centrifuged at 4 °C and 12,000×g for 30 min. After careful removal of the supernatant, the resulting pellet was resuspended in sterile water and mixed thoroughly. The suspension was centrifuged again, and the supernatant was collected to extract water-soluble polysaccharides. The obtained precipitate was dispersed in 0.1 mol/L sodium hydroxide solution, centrifuged at 4 °C and 12,000× *g* for 30 min. The supernatant was collected, and three volumes of 95% ethanol were added, followed by incubation at 4 °C overnight. Subsequently, the alkali-soluble polysaccharide precipitate was collected by centrifugation. The content of extracellular polysaccharides was determined using the phenol–sulfuric acid method.

### 2.8. Real-Time Quantitative PCR Analysis of Gene Expression Levels

The effect of CAPE on the expression levels of biofilm-related genes in MRSA CI2 was detected by real-time quantitative PCR (RT-qPCR) [30]. Briefly, the MRSA culture was centrifuged at 4 °C and 12,000×g for 30 min. After collecting the precipitate, it was incubated with 16 µg/mL CAPE at 37 °C for 24 h. Total RNA was extracted using the flying shark plus bacteria RNA Kit (Nobelab, Jinan, China), and RNA was reverse-transcribed into cDNA using the ALL-in-One First-Strand Synthesis MasterMix (LABLEAD, Beijing, China) according to the instructions. RT-qPCR amplification of the cDNA template was performed using 2 × Realab Green PCR Fast Mixture (LABLEAD, Beijing, China), and the experiment was completed on the QuantStudio platform of Applied Biosystems (Waltham, MA, USA). The gene expression level was calculated using the 2^−ΔΔCt^ method, and the threshold cycle number was normalized using 16S rDNA as the reference gene. The primer sequences are detailed in Table 1.

### 2.9. Statistical Analysis

All experiments were carried out in triplicate independently. Results are shown as mean ± standard deviation. GraphPad Prism 10 was used for statistical analysis. Student’s *t*-test was employed for comparisons between two groups, while one-way analysis of variance (ANOVA) followed by Tukey’s multiple comparisons test was applied for comparisons among multiple groups. Statistical significance was set at *p* < 0.05.

## 3. Results

### 3.1. MIC of CAPE

**Figure 1:**
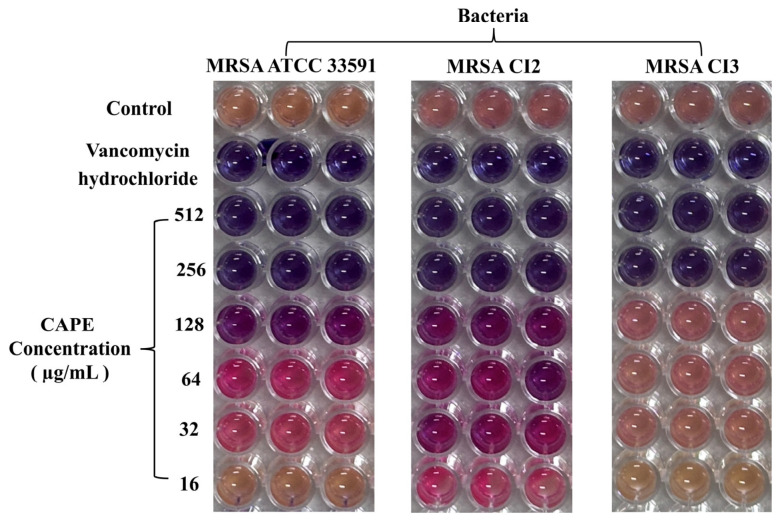
Schematic representation of the MIC values of CAPE against MRSA strains.

The MIC of CAPE against all strains was assessed using the broth microdilution method. Table 2 and Figure 1 show that the MIC values of CAPE against three MRSA strains were 256 µg/mL, indicating that CAPE showed antimicrobial activity against MRSA.

### 3.2. Effect of CAPE Against MRSA Biofilms Formation

**Figure 2:**
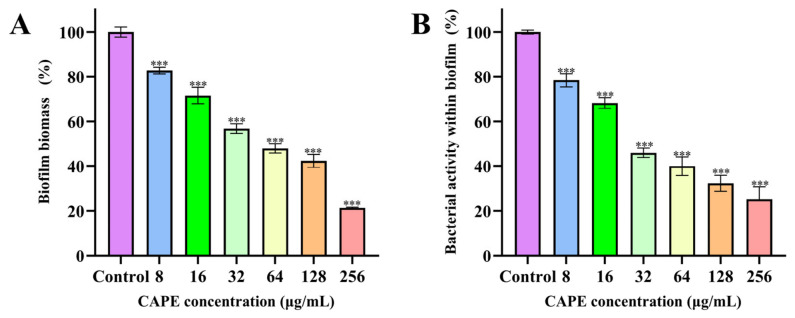
Effects of CAPE on biofilm formation of MRSA CI2. (**A**) biofilm biomass assessed by CV assay; (**B**) biofilm metabolic activity assessed by XTT assay. All data are presented as mean values ± SD, and *n* = 3 in each group. *** *p* < 0.001, vs. control.

To investigate the effect of CAPE on the formation of MRSA biofilms, the biomass and metabolic activity of the biofilm were measured. As shown in Figure 2, when CAPE was applied at 8 µg/mL or higher, the biomass and metabolic activity of MRSA biofilms showed a significant decreasing trend compared with the control group. These findings indicate that CAPE strongly inhibits the establishment of fresh MRSA biofilms.

### 3.3. Effect of CAPE Against Mature MRSA Biofilms

**Figure 3:**
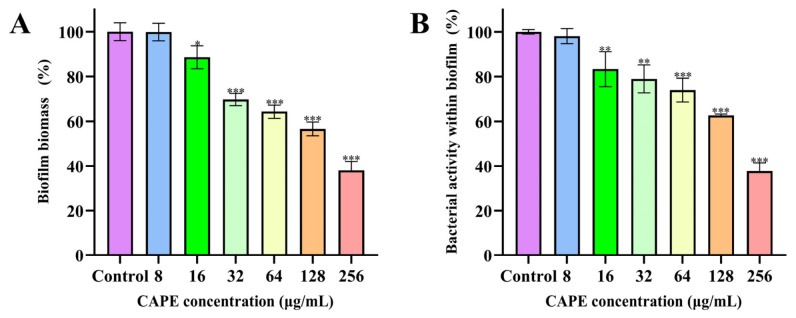
Effects of CAPE on mature biofilm of MRSA CI2. (**A**) biofilm biomass assessed by CV assay; (**B**) biofilm metabolic activity assessed by XTT assay. All data are presented as mean values ± SD, and *n* = 3 in each group. * *p* < 0.05, ** *p* < 0.01, *** *p* < 0.001, vs. control.

To explore how CAPE affects established MRSA biofilms, this study quantified both biomass and metabolic activity of the biofilm. In Figure 3, treatment with CAPE at concentrations of 16 µg/mL or higher significantly reduced both biomass and metabolic activity of MRSA biofilms compared to the control group. This concentration-dependent inhibitory effect indicates that CAPE exhibits excellent antibiofilm activity against mature MRSA biofilms.

### 3.4. Effect of CAPE on the Structure of Biofilms

**Figure 4:**
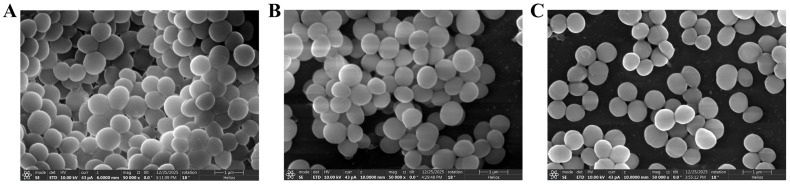
Effects of different concentrations of CAPE on the overall structure of MRSA CI2 biofilms. (**A**) Photograph of the biofilm structure of MRSA CI2 not treated by CAPE; (**B**) photograph of the biofilm structure of MRSA CI2 treated with 64 µg/mL of CAPE; (**C**) photograph of the biofilm structure of MRSA CI2 treated with 128 µg/mL of CAPE.

The overall structural integrity of MRSA biofilms following CAPE treatment was visualized by SEM. In Figure 4, MRSA biofilms in the CAPE-treated group differed markedly from those in the untreated group. As shown in Figure 4A, MRSA cells in the control group were closely packed, forming a dense biofilm where bacterial cells adhered to each other. However, as shown in Figure 4B,C, when CAPE was applied at concentrations of 64 µg/mL and 128 µg/mL, no obvious damage to the overall cell structure was observed; nevertheless, the biofilm structure became loose, and adhesion and aggregation were significantly reduced. According to these findings, CAPE suppressed both bacterial adhesion and aggregation of MRSA while also decreasing the development of biofilms.

### 3.5. Effect of CAPE on Bacterial Adhesion

**Figure 5:**
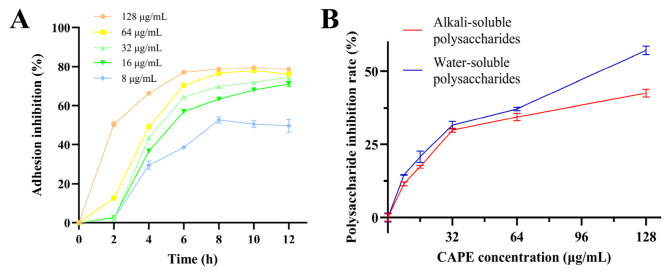
Effects of CAPE on MRSA bacterial adhesion and extracellular polysaccharide production. (**A**) CAPE inhibits the adhesion of MRSA CI2; (**B**) CAPE inhibits the production of extracellular polysaccharides in MRSA CI2. All data are presented as mean values ± SD, and *n* = 3 in each group.

The adhesion of bacteria is essential for starting the biofilm development process as well as for maintaining already-established biofilms. Therefore, the adhesion ability of bacteria can indirectly reflect their potential to form biofilms [24]. As shown in Figure 5, CAPE significantly inhibits MRSA adhesion in a concentration-dependent manner. The experimental results indicate that CAPE can effectively inhibit the adhesion of MRSA and reduce the density of biofilms. Thus, the reduction of bacterial adhesion by CAPE may represent a key mechanism underlying its anti-MRSA biofilm.

### 3.6. Effect of CAPE on Extracellular Polysaccharide Production

Alkali-soluble polysaccharides contribute to MRSA adhesion and subsequent biofilm maturation via their adhesive properties, whereas water-soluble polysaccharides offer nutritional support essential for MRSA colonization [28,34]. As shown in Figure 5, treatment with CAPE at concentrations of 8 to 128 µg/mL resulted in concentration-dependent decreases in both alkali-soluble and water-soluble polysaccharide levels in MRSA. Collectively, these data confirm that CAPE can effectively inhibit the polysaccharide synthesis of MRSA.

### 3.7. Effect of CAPE on Adhesion-Related Gene Expression

**Figure 6:**
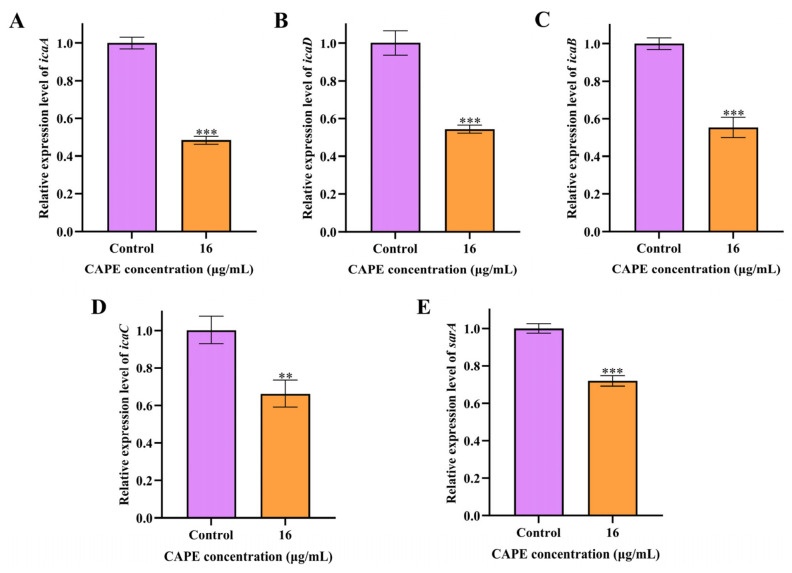
Effects of CAPE on the biofilm gene expression of MRSA CI2.  (**A**) CAPE inhibit the expression of *icaA*; (**B**) CAPE inhibit the expression of *icaD*; (**C**) CAPE inhibit the expression of *icaB*; (**D**) CAPE inhibit the expression of *icaC*; (**E**) CAPE inhibit the expression of *sarA.* All data are presented as mean values ± SD, and *n* = 3 in each group. ** *p* < 0.01, *** *p* < 0.001, vs. control.

**Figure 7:**
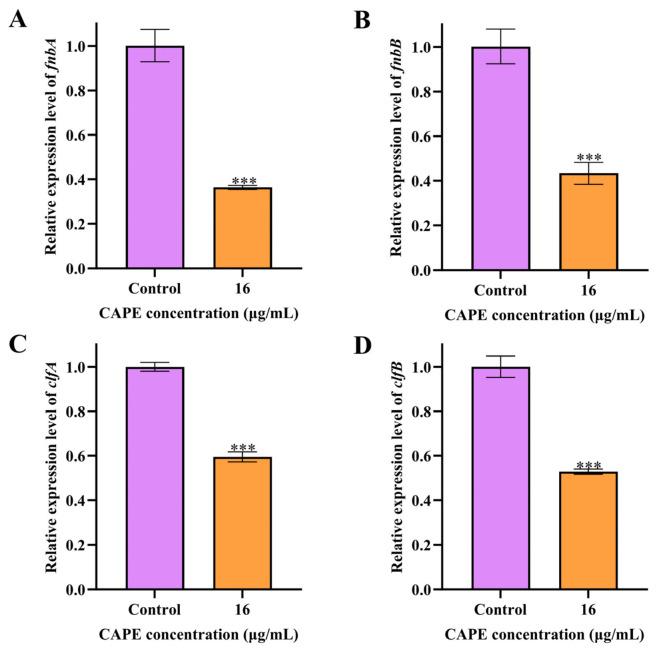
Effects of CAPE on the biofilm gene expression of MRSA CI2. (**A**) CAPE inhibit the expression of *fnbA*; (**B**) CAPE inhibit the expression of *fnbB*; (**C**) CAPE inhibit the expression of *clfA; *(**D**) CAPE inhibit the expression of *clfB*. All data are presented as mean values ± SD, and *n* = 3 in each group. *** *p* < 0.001, vs. control.

This study analyzed the impact of CAPE on the expression levels of adhesion-related genes for MRSA PIA (polysaccharide intercellular adhesin, the major exopolysaccharide) synthesis (*icaADBC*), fibronectin-binding protein regulatory genes (*fnbAB*), clumping factor genes (*clfAB*), and a regulatory factor (*sarA*). For MRSA CI2, CAPE significantly downregulated the expression levels of *icaADBC* and *sarA* in Figure 6. Similarly, CAPE significantly downregulated the expression levels of *fnbAB* and *clfAB* in Figure 7.

## 4. Discussion

MRSA is a multidrug-resistant and highly pathogenic bacterium that exhibits potent resistance to β-lactam antibiotics. It poses a transmission risk in both hospital and community environments, demonstrating significant clinical harm and spreading capability [35,36]. In addition, existing studies have confirmed that the key inducement of malignant infections caused by MRSA is closely related to the presence of biofilms. This structure can not only help bacteria achieve stable surface colonization but also protect them from elimination by the host’s immune system and antibiotics [37]. Moreover, the formation of biofilms will further enhance the resistance of MRSA, greatly reducing the therapeutic effect of antibacterial drugs. CAPE, a natural phenolic compound predominantly derived from propolis, exhibits a broad spectrum of biological activities. Previous studies have shown that CAPE exhibits significant antibacterial and antibiofilm activities against a variety of oral microorganisms, such as *Staphylococcus aureus*, *Streptococcus mutans*, and *Streptococcus salivarius* [22,38]. However, no relevant studies on the antibiofilm activity and underlying mechanisms of CAPE against MRSA have been reported to date; therefore, our findings cannot be directly compared with the existing literature. In this study, despite the relatively high MIC values of CAPE against planktonic MRSA strains, its antibiofilm activity was remarkably potent, especially against CI2, a strain characterized by strong biofilm formation. The results showed that CAPE can not only block the initial formation process of fresh MRSA biofilms but also disrupt the development and consolidation of biofilms. Furthermore, SEM observations indicated that MRSA biofilms treated with CAPE appeared loose and fragmented, and the integrity of the bacterial colonies was destroyed. These findings are consistent with CAPE’s ability to impair biofilm adhesion.

The formation of MRSA biofilms is a complex process with multiple stages and precise regulation by various factors. The formation of biofilms follows the law of dynamic evolution, mainly going through four consecutive stages: adhesion, aggregation and proliferation, maturation, and detachment and diffusion. Among these stages, microbial adhesion represents a crucial stage, as it not only enables the assembly of newly forming biofilms but also contributes to maintaining the integrity of those already present [39,40]. Based on this, this study systematically explored how CAPE influences MRSA adhesion. The experimental results showed that CAPE had a significant inhibitory impact on MRSA adhesion. The adhesion ability of MRSA is closely related to the synthesis of its extracellular polysaccharides. The results of this study indicated that CAPE demonstrated a strong ability to suppress the production of both alkali- and water-soluble polysaccharides in MRSA. This suggests that the above-mentioned inhibition of the synthesis of extracellular polysaccharides may be one of the effective mechanisms by which CAPE blocks the adhesion process of MRSA.

Polysaccharide intercellular adhesin (PIA) is the main extracellular polysaccharide, and its synthesis is regulated by the *icaADBC* genes [30]. PIA synthesis is catalytically mediated by proteins encoded by the ica operon, which consists of four functional genes (*icaADBC*) and a repressor gene (*icaR*). Among them, the protein encoded by *icaA* has N-acetylglucosamine transferase activity, the expression product of *icaD* can significantly enhance this enzyme activity, the *icaB* gene is responsible for encoding a secretory deacetylase, and the membrane protein encoded by *icaC* is responsible for the extracellular transport of PIA [41]. *icaR* is transcribed in the reverse orientation relative to *icaADBC*, and the product resulting from its expression acts to suppress the activity of this genetic unit [42].

Furthermore, it has been reported that fibronectin-binding proteins (FnbpA, FnbpB), clumping factors (ClfA, ClfB), fibrinogen-binding protein (Fib), laminin-binding protein (Eno), elastin-binding protein (Ebps), collagen-binding protein (Cna), and extracellular adhesion protein (Eap) can promote the adhesion and aggregation of bacteria, thereby supporting the formation of biofilms, with the corresponding genes being *fnbAB*, *clfAB*, *fib*, *eno*, *ebpS*, *cna*, *bbp* and *eap* [43,44,45]. The DNA-binding protein SarA, encoded by *sarA*, plays a critical role in *ica* operon transcription, PIA synthesis, and biofilm formation. It is a positive regulatory factor for the functional genes of the *ica* operon and an important global regulatory factor for various staphylococcal virulence factors [46]. Having established that CAPE markedly suppresses expression of the ica gene, which regulates PIA production in MRSA, the present investigation subsequently examined how this compound affects additional genetic regulators responsible for the previously mentioned proteins within MRSA. We found that CAPE can significantly reduce the expression of the *icaADBC*, *sarA*, *fnbAB*, and *clfAB* genes in MRSA CI2. Based on the above results, it is speculated that the potential mechanism of action of CAPE involves two pathways: one leading to significant inhibition of PIA synthesis, and the other resulting in reduced production of various adhesion-related proteins. The potential mechanism of action may be the key to CAPE’s inhibition of MRSA biofilm formation. Furthermore, previous studies from our group have shown that propolis displays marked antibiofilm activity against MRSA [29]. Since CAPE is a major constituent of propolis, the current findings lead us to propose that CAPE is one of the principal bioactive compounds responsible for the antibiofilm properties of propolis. Furthermore, given that CAPE did not completely inhibit MRSA biofilms, future studies could focus on its combination with conventional antibiotics as a potential strategy to improve overall biofilm inhibitory efficacy. Overall, CAPE exhibits significant inhibitory activity against MRSA biofilms, and this property indicates that the substance has broad application potential in the treatment of MRSA-mediated bacterial infections.

## 5. Conclusions

This study found that CAPE has significant antibiofilm activity against MRSA. Its antibiofilm effects are mainly manifested as: significantly reducing the biofilms’ biomass and decreasing biofilm activity. By detecting bacterial adhesion ability, extracellular polysaccharides, and the expression of related genes, this study clarified the possible mechanism by which CAPE inhibits MRSA biofilm formation: CAPE downregulates the expression of the *icaADBC*, *sarA*, *fnbAB*, and *clfAB* genes, thereby reducing the synthesis of extracellular polysaccharide and adhesion-related proteins, weakening the adhesion ability of MRSA, and consequently achieving antibiofilm effects. In conclusion, CAPE has broad application prospects in the prevention and treatment of MRSA-related diseases.

## Figures and Tables

**Table 1:** Primers employed in this work.

Gene	Primer Sequences (5′→3′)	Reference
*icaA*	F: CTTGCTGGCGCAGTCAATAC	[31]
	R: GTAGCCAACGTCGACAACTG	
*ica* *B*	F: CCTGTAAGCACACTGGATGG	[31]
	R: TCGCTTTTCTTACACGGTGA	
*ica* *C*	F: TGCGTTAGCAAATGGAGACT	[31]
	R: TGCGTGCAAATACCCAAGAT	
*ica* *D*	F: TGGGCATTTTCGCGATTATCA	[31]
	R: ACGATTCTCTTCCTTTCTGCCA	
*sarA*	F: GTAATGAGCATGATGAAAGAACTGT	[31]
	R: CGTTGTTTGCTTCAGTGATTCG	
*fnbA*	F: AAATTGGGAGCAGCATCAGT	[32]
	R: GCAGCTGAATTCCCATTTTC	
*fnbB*	F: CAACCAGTCGTTAAGCTCTGTGAC	[33]
	R: GCTGACATCATCAAGCTTTGC	
*clfA*	F: ACCCAGGTTCAGATTCTGGCAGCG	[32]
	R: TCGCTGAGTCGGAATCGCTTGCT	
*clfB*	F: ATCATCAAGCACACGCATCA	
	R: TGCAGCATTTACTACCGGTT	
*16S rRNA*	F: CGCAATGGGCGAAAGC	[31]
	R: TACGATCCGAAGACCTTCATCA	

**Table 2:** MIC values of CAPE against MRSA.

Compound	Bacteria	MIC (µg/mL)
CAPE	MRSA ATCC 33591	256
	MRSA CI2	256
	MRSA CI3	256

## Data Availability

The original contributions presented in this study are included in the article. Further inquiries can be directed to the corresponding author.

## Abbreviations

The following abbreviations are used in this manuscript:

MRSA	Methicillin-Resistant *Staphylococcus aureus*
PBP 2a	Penicillin-Binding Protein 2a
EPS	Extracellular Polymeric Substance
CAPE	Caffeic Acid Phenethyl Ester
MIC	Minimal Inhibitory Concentration
TSB	Trypticase Soy Broth
PBS	Phosphate-Buffered Saline
CFU	Colony-Forming Unit
CV	Crystal Violet
XTT	2,3-Bis-(2-methoxy-4-nitro-5-sulfophenyl)-2H-tetrazolium-5-carboxanilide
SEM	Scanning Electron Microscopy
RT-qPCR	Real-time quantitative Polymerase Chain Reaction
PIA	Polysaccharide Intercellular Adhesin

## References

[1] Liu C., Bayer A., Cosgrove S.E., Daum R.S., Fridkin S.K., Gorwitz R.J., Kaplan S.L., Karchmer A.W., Levine D.P., Murray B.E. (2011). Clinical Practice Guidelines by the Infectious Diseases Society of America for the Treatment of Methicillin-Resistant *Staphylococcus Aureus* Infections in Adults and Children. Clin. Infect. Dis..

[2] Turner N.A., Sharma-Kuinkel B.K., Maskarinec S.A., Eichenberger E.M., Shah P.P., Carugati M., Holland T.L., Fowler V.G. (2019). Methicillin-resistant *Staphylococcus aureus*: An overview of basic and clinical research. Nat. Rev. Microbiol..

[3] Beker S., Demirbilek S.K. (2025). Optimizing detection methods for MRSA isolated from mastitis cases and assessing virulence genes. Res. Vet. Sci..

[4] Guo Y., Ding L., Han R.R., Yin D.D., Wu S., Yang Y., Wang F., Zhu D.M., Hu F.P., the China Antimicrobial Surveillance NetworkStudy Group (2025). Antimicrobial resistance profile of clinical isolates from hospitals across China: CHINET 2024 surveillance report. One Health Adv..

[5] Qi M., Liu Q., Liu Y., Yan H., Zhang Y., Yuan Y. (2022). *Staphylococcus aureus* biofilm inhibition by high voltage prick electrostatic field (HVPEF) and the mechanism investigation. Int. J. Food Microbiol..

[6] Uruén C., Chopo-Escuin G., Tommassen J., Mainar-Jaime R.C., Arenas J. (2020). Biofilms as Promoters of Bacterial Antibiotic Resistance and Tolerance. Antibiotics.

[7] Prosswimmer T., Nick S.E., Bryers J.D., Daggett V. (2024). Designed De Novo α-Sheet Peptides Destabilize Bacterial Biofilms and Increase the Susceptibility of *E. coli* and *S. aureus* to Antibiotics. Int. J. Mol. Sci..

[8] Behera S., Mumtaz S., Singh M., Mukhopadhyay K. (2023). Synergistic Potential of α-Melanocyte Stimulating Hormone Based Analogues with Conventional Antibiotic against Planktonic, Biofilm-Embedded, and Systemic Infection Model of MRSA. ACS Infect. Dis..

[9] El-Shiekh R.A., Radi M.H., Elshimy R., Abdel-Sattar E., El-Halawany A.M., Ibrahim M.A., Ali M.E., Hassanen E.I. (2025). Friedelin: A natural compound exhibited potent antibacterial, anti-inflammatory, and wound healing properties against MRSA-infected wounds. Naunyn-Schmiedeberg’s Arch. Pharmacol..

[10] Craft K.M., Nguyen J.M., Berg L.J., Townsend S.D. (2019). Methicillin-resistant *Staphylococcus aureus* (MRSA): Antibiotic-resistance and the biofilm phenotype. MedChemComm.

[11] Tolba M.F., Azab S.S., Khalifa A.E., Abdel-Rahman S.Z., Abdel-Naim A.B. (2013). Caffeic acid phenethyl ester, a promising component of propolis with a plethora of biological activities: A review on its anti-inflammatory, neuroprotective, hepatoprotective, and cardioprotective effects. IUBMB Life.

[12] Jung W.K., Lee D.Y., Kim J.H., Choi I., Park S.G., Seo S.K., Lee S.W., Lee C.M., Park Y.M., Jeon Y.J. (2008). Anti-inflammatory activity of caffeic acid phenethyl ester (CAPE) extracted from Rhodiola sacra against lipopolysaccharide-induced inflammatory responses in mice. Process Biochem..

[13] Gölcük V.A., Şeneldir L. (2025). The effect of caffeic acid phenethyl ester on facial nerve regeneration. Acta Oto-Laryngol..

[14] Aquino I.G., de Almeida P.C., Rangel-Coelho J.P., Raucci L.M.S.D.C., Martinez E.F., Teixeira L.N. (2025). Influence of Caffeic Acid Phenethyl Ester on Osteoblastic Cell Behavior in Coculture with Breast Adenocarcinoma Cells. Anticancer Res..

[15] Lin T.P., Chen P.C., Lin C.Y., Wang B.J., Kuo Y.Y., Yeh C.C., Tseng J.C., Huo C., Kao C.L., Shih L.J. (2025). Prostate cancer cells elevate glycolysis and G6PD in response to caffeic acid phenethyl ester-induced growth inhibition. BMC Cancer.

[16] Lim S.C., Lee T.B., Han S.I. (2025). Caffeic Acid Phenethyl Ester Inhibits Metastatic Properties of Acid-adapted Gastric Cancer Cells. Anticancer Res..

[17] Sapmaz H.I., Köse E., Karaca Z.M., Akyürek S., Parlakpinar H., Deresoy F.A., Türköz Y., Dağli A.F. (2025). Investigating the possible protective effect of caffeic acid phenethyl ester on aquaporin-2 changes in renal ischemia-reperfusion injury in rats. Cir. Y Cir..

[18] Suningdyastiningrum A.O., Hutami I.R., Berliani Y., Rochmah Y.S. (2025). Caffeic acid phenethyl ester promotes palatal wound healing and enhances wound-associated macrophage CD68 expression. J. Taibah Univ. Med. Sci..

[19] Sulimai N., Brown J., Lominadze D. (2025). Caffeic Acid Phenethyl Ester Protects Neurons Against Oxidative Stress and Neurodegeneration During Traumatic Brain Injury. Biomolecules.

[20] Arasoglu T., Derman S., Mansuroglu B. (2016). Comparative evaluation of antibacterial activity of caffeic acid phenethyl ester and PLGA nanoparticle formulation by different methods. Nanotechnology.

[21] Chen X., Yang M., Qin J.J., Wu Z.S., Ji W. (2025). Coassembly of Betulinol with Glycyrrhetinic Acid or Caffeic Acid Phenethyl Ester and Their Characteristics. ACS Omega.

[22] Kokilakanit P., Dungkhuntod N., Serikul N., Koontongkaew S., Utispan K. (2025). Caffeic acid phenethyl ester inhibits multispecies biofilm formation and cariogenicity. PeerJ.

[23] Wang F., Yuan J., Wang X.R., Xuan H.Z. (2023). Antibacterial and anti-biofilm activities of Chinese Propolis essential oil microemulsion against *Streptococcus mutans*. J. Appl. Microbiol..

[24] Sang H., Jin H., Song P., Xu W., Wang F. (2024). Gallic acid exerts antibiofilm activity by inhibiting methicillin resistant *Staphylococcus aureus* adhesion. Sci. Rep..

[25] Navarro-Pérez M.L., Vadillo-Rodríguez V., Fernández-Babiano I., Pérez-Giraldo C., Fernández-Calderón M.C. (2021). Antimicrobial activity of a novel Spanish propolis against planktonic and sessile oral *Streptococcus* spp.. Sci. Rep..

[26] Wang F., Wei F.Y., Song C.X., Jiang B., Tian S.Y., Yi J.W., Yu C.L., Song Z.B., Sun L.G., Bao Y.L. (2017). *Dodartia orientalis* L. essential oil exerts antibacterial activity by mechanisms of disrupting cell structure and resisting biofilm. Ind. Crops Prod..

[27] Niu Y.M., Wang K., Zheng S.N., Wang Y.F., Ren Q., Li H.R., Ding L.J., Li W., Zhang L.L. (2020). Antibacterial effect of caffeic acid phenethyl ester on cariogenic bacteria and *Streptococcus mutans* biofilms. Antimicrob. Agents Chemother..

[28] Liu J., Li W., Zhu X.Y., Zhao H.Z., Lu Y.J., Zhang C., Lu Z.X. (2019). Surfactin effectively inhibits *Staphylococcus aureus* adhesion and biofilm formation on surfaces. Appl. Microbiol. Biotechnol..

[29] Sang H., Feng K.Y., Ju Y.H., Sun Y.Y., Zhang Y., Xuan H.Z., Wang F. (2025). Propolis Exerts Antibiofilm Activity Against Methicillin-Resistant *Staphylococcus aureus* by Modulating Gene Expression to Suppress Adhesion. Microorganisms.

[30] Feng K.Y., Sang H., Jin H., Song P., Xu W., Xuan H.Z., Wang F. (2025). Antimicrobial Activities of Propolis Nanoparticles in Combination with Ampicillin Sodium Against Methicillin-Resistant *Staphylococcus aureus*. Microorganisms.

[31] Zhang W.W., Margarita G.E., Wu D., Yuan W.Q., Yan S., Qi S.Z., Xue X.F., Wang K., Wu L.M. (2022). Antibacterial Activity of Chinese Red Propolis against *Staphylococcus aureus* and MRSA. Molecules.

[32] Mastoor S., Nazim F., Hasan S.R., Ahmed K., Khan S., Ali S.N., Abidi S.H. (2022). Analysis of the Antimicrobial and Anti-Biofilm Activity of Natural Compounds and Their Analogues against *Staphylococcus aureus* Isolates. Molecules.

[33] Xiong Y.Q., Sharma-Kuinkel B.K., Casillas-Ituarte N.N., Fowler V.G., Rude T., DiBartola A.C., Lins R.D., Abdel-Hady W., Lower S.K., Bayer A.S. (2015). Endovascular infections caused by methicillin-resistant *Staphylococcus aureus* are linked to clonal complex-specific alterations in binding and invasion domains of fibronectin-binding protein A as well as the occurrence of *fnbB*. Infect. Immun..

[34] Rohde H., Burandt E.C., Siemssen N., Frommelt L., Burdelski C., Wurster S., Scherpe S., Davies A.P., Harris L.G., Horstkotte M.A. (2007). Polysaccharide intercellular adhesin or protein factors in biofilm accumulation of *Staphylococcus epidermidis* and *Staphylococcus aureus* isolated from prosthetic hip and knee joint infections. Biomaterials.

[35] Yao S.T., Fang C., Xu B.J., Hu Y., Chen Z., Xue X.Y., Liu J.P., Li M.K., Li P.Y. (2025). Designing novel nucleoside inhibitors targeting the allosteric site of PBP2a: A strategic approach to overcome resistance in MRSA. Bioorganic Med. Chem..

[36] Ripari N., Pereira A.F.M., Júnior A.F., Rall V.L.M., Aldana-Mejía J.A., Bastos J.K., Sforcin J.M. (2023). Brazilian red propolis in combination with β-lactams exerts an efficient antibacterial action over methicillin-resistant *Staphylococcus aureus* (MRSA) strains. J. Appl. Microbiol..

[37] Kaushik A., Kest H., Sood M., Steussy B.W., Thieman C., Gupta S. (2024). Biofilm Producing Methicillin-Resistant *Staphylococcus aureus* (MRSA) Infections in Humans: Clinical Implications and Management. Pathogens.

[38] AlSheikh R., Albagieh H.N., Abdouh I., Zaki H., Alzahrani A.M., Halawany H.S., Al-Khalifa K.S. (2022). In Vitro Activity of Caffeic Acid Phenethyl Ester against Different Oral Microorganisms. Appl. Sci..

[39] Palmer J., Flint S., Brooks J. (2007). Bacterial cell attachment, the beginning of a biofilm. J. Ind. Microbiol. Biotechnol..

[40] Liu R., Memarzadeh K., Chang B., Zhang Y.M., Ma Z., Allaker R.P., Ren L., Yang K. (2016). Antibacterial effect of copper-bearing titanium alloy (Ti-Cu) against *Streptococcus mutans* and *Porphyromonas gingivalis*. Sci. Rep..

[41] Lister J.L., Horswill A.R. (2014). *Staphylococcus aureus* biofilms: Recent developments in biofilm dispersal. Front. Cell. Infect. Microbiol..

[42] Arciola C.R., Campoccia D., Ravaioli S., Montanaro L. (2015). Polysaccharide intercellular adhesin in biofilm: Structural and regulatory aspects. Front. Cell. Infect. Microbiol..

[43] Vergara-Irigaray M., Valle J., Merino N., Latasa C., Garcia B., De Los Mozos I.R., Solano C., Toledo-Arana A., Penades J.R., Lasa I. (2009). Relevant role of fibronectin-binding proteins in *Staphylococcus aureus* biofilm-associated foreign-body infections. Infect. Immun..

[44] Kot B., Sytykiewicz H., Sprawka I., Witeska M. (2020). Effect of manuka honey on biofilm-associated genes expression during methicillin-resistant *Staphylococcus aureus* biofilm formation. Sci. Rep..

[45] Wcisłek A., Jursa-Kulesza J., Masiuk H., Grygorcewicz B., Hukowska-Szematowicz B., Prowans P., Ziętek P., Kosik-Bogacka D. (2025). Phenotypic and Genotypic Characterization of *Staphylococcus aureus* Isolated from Patients with Chronic Furunculosis and Osteomyelitis from Northwestern Poland. Pathogens.

[46] Deng X.B., Xu H.B., Li D.Y., Chen J.L., Yu Z.J., Deng Q.W., Li P.Y., Zheng J.X., Zhang H.G. (2023). Mechanisms of Rapid Bactericidal and Anti-Biofilm Alpha-Mangostin In Vitro Activity against *Staphylococcus aureus*. Pol. J. Microbiol..

